# Effects of cannabinoid and vanilloid receptor antagonists on nicotine induced relaxation response enhancement in rabbit corpus cavernosum

**DOI:** 10.22038/IJBMS.2022.62222.13772

**Published:** 2022-04

**Authors:** Ismail Mert Vural, Gokce Sevi Ozturk Fincan, Derya Sebile Koc, Yagmur Okcay, Celil Ilker Askin, Ayse Kubra Kibar, Sevil Ozger Ilhan, Yusuf Sarioglu

**Affiliations:** 1 Department of Pharmacology, Gulhane Faculty of Pharmacy, University of Health Sciences Turkey, Ankara, Turkey; 2 Department of Medical Pharmacology, School of Medicine, Gazi University, Ankara, Turkey; 3 Department of Medical Pharmacology, School of Medicine, Istinye University, Istanbul, Turkey

**Keywords:** Cannabinoid receptors, Endocannabinoids, Nicotine, Penile erection, Smooth muscle

## Abstract

**Objective(s):**

Endocannabinoids and nicotine regulate the neurotransmitter release in different central and peripheral synapses. Various studies in the literature demonstrate the interaction between endocannabinoid and nicotinic systems, especially in the central nervous system. The interaction between nicotinic and endocannabinoid systems was investigated in this study. We aimed to show the effects of cannabinoid and vanilloid receptor antagonists on nicotine-induced relaxation response increases in rabbit corpus cavernosum.

**Materials and Methods:**

From a total of seven male albino rabbits, three or four equal strips were cut from each corpus cavernosum and inserted in isolated organ baths. Tissues were contracted with phenylephrine (3×10^−5^ M). After contraction reached a plateau, strips were stimulated with EFS, and with the stabilization of EFS relaxation responses, 10^-4 ^M of nicotine was administered to tissues. After that, in order to investigate the effects of AM251 (CB_1_ antagonist), AM630 (CB_2_ inverse agonist) or capsazepine (a vanilloid receptor antagonist) were given to different tissues, after the resting period.

**Results:**

Nicotine (10^−4^ M) increased the EFS-induced relaxation responses (14.60%±2.94%, *P*<0.05). AM630 decreased the enhancement of nicotine-induced EFS relaxation responses (nicotine 10^-4^ M enhancement: 17.16%±3.19%; nicotine 10^-4^ M enhancement in the presence of AM630 10^-6 ^M: 4.44%±3.43% *P*<0.05; n=6), whereas effects of AM251 and capsazepine were not significant.

**Conclusion:**

In the present study, nicotine increased the amplitudes of EFS-induced relaxation responses probably via nicotinic acetylcholine receptors located on the nitrergic nerves of the corpus cavernosum. We showed the role of cannabinoid-like endo-ligands in nicotine-induced enhancement via CB_2_ receptors but not CB_1_ and VR_1_ receptors.

## Introduction

Relaxation of corpus cavernosum smooth muscle is essential for penile erection. In the corpus cavernosum, smooth muscle Nitric oxide (NO) is the primary mediator of relaxation responses (1). NO increases the production of cyclic guanosine monophosphate (cGMP) via the soluble guanylate cyclase enzyme (2). In our previous studies, we showed that L-arginine methyl ester (L-NAME) nearly abolished Electrical Field Stimulation (EFS)-induced relaxation of the corpus cavernosum at low frequencies (3-5). In one of those studies, we concluded that nicotine via nicotinic acetylcholine receptors (nAChR) which were located on the nitrergic nerves evoked the release of NO in the corpus cavernosum tissue of the rabbit (3). nAChRs are important ligand-gated ion channels that modulate neurotransmitter release in central and peripheral synapses (6-9). Like acetylcholine and other cholinergic ligands, nicotine is a ligand of these receptors (9). Previously we also showed the enhancement effect of nicotine on smooth muscle responses via EFS in various peripheral tissues other than the corpus cavernosum (10-12). The endocannabinoid system consists of endogenous ligands, cannabinoid receptors (CBRs), synthesizing and inactivating enzymes, and a transport protein (13). Endocannabinoid ligands through presynaptic cannabinoid receptors modulate neurotransmitter release in various synapses of different systems (14-17). It has been indicated that endocannabinoids reduce neurotransmitter release in various central neuron synapses via presynaptic CB_1_ receptors (18). CB_2_ receptors and their functions have been demonstrated in many studies, especially in the immune system and other peripheral tissues (19-21). Our previous studies demonstrated that endocannabinoids might have a role in EFS-evoked responses through CB_1_ and CB_2_ receptors of the rabbit corpus cavernosum (5) and CB_1_ receptors in the rabbit vas deferens (22). Ghasemi *et al*. (2006) showed that anandamide, an endogenous non-selective cannabinoid receptor agonist, has enhanced nonadrenergic noncholinergic (NANC) relaxation responses in the corpus cavernosum of the rat. They also detected CB_1_ and VR_1_ (a vanilloid receptor) receptor proteins in tissue (23). Cannabinoid receptor’s presence in human oocytes was also shown in a different study (24). 

Interaction between endocannabinoid and nicotinic systems has been shown in different studies, especially in the central nervous system (25-28). Pekala *et al*. (2018) have shown the role of cannabinoid receptors on stress- and nicotine-related behavioral changes in mice (25). It was demonstrated that some nAChR subtypes are associated with cannabis disorders and they might be a target for tetrahydrocannabinol (THC) dependence treatment (26). Moreover, the effects of the CB_1_ cannabinoid receptor in the basolateral amygdala on nicotine-induced responses have been previously demonstrated (28). There are few studies on the interaction between these two systems in peripheral tissues. In Xenopus oocytes, it was demonstrated that anandamide, the endogenous cannabinoid, inhibited the function of alpha 7 subtypes of nAChR without interacting with cannabinoid receptors (29). The interaction between these two systems has not yet been investigated in the corpus cavernosum tissue. Finding the effects of this interaction on smooth muscle relaxation responses will be beneficial both in terms of elucidating the relaxation physiology and identifying possible treatment target candidates. 

As mentioned above, in our previous studies, we demonstrated the effect of nicotine on nitrergic relaxation responses via nAChRs (3, 4). In this study, we investigate the interaction between nicotinic and endocannabinoid systems. We aimed to show the effects of cannabinoid as well as vanilloid receptor antagonists on nicotine-induced EFS relaxation response increase in rabbit corpus cavernosum.

## Materials and Methods

This study is conducted methodologically similar to our previous studies, which involved the investigation of nicotine’s effect on EFS responses in rabbit corpus cavernosum (8-9). Tissues were prepared as previously described in the literature (30). Gazi University Ethics Committee approved this study for animal experimentation (G.U.E.T-21.019). All experiments were done in the first half of June 2021. 

***Animals and preparation of isolated corpus cavernosum tissues***

A total of seven male albino New Zealand rabbits were used. Rabbits were 2–3 months old and their weights were between 2.5 and 3.0 kg. Animals had free access to standard laboratory chow and tap water *ad libitum*. All animals were sacrificed in the morning. Then the experimental protocol was completed in the afternoon of the same day. Animals were anesthetized via the ear vein with thiopental (%50 mg/kg, IV) and after the anesthesia, the penis was isolated from where the corpus cavernosum is connected to the ischium. The tissue was kept in Krebs solution, was heated to 37 °C, then the tissue was aired with a combination of 95% O_2 _and 5% CO_2_. Afterward, the penile tissue was cleaned from the urethra, connective tissue, corpus spongiosum, and epidermal tissue. Three or four equal strips of 3x3x15 mm were cut from each corpus cavernosum according to the corpus cavernosum size. Each strip was studied separately. The prepared strips were placed in a 20 ml organ bath which contained Krebs solution via organ hoop as one end of the strip was tied to the organ hoop and the other end was connected to an isometric power transducer (MAY, FDT 10**‐**A, COMMAT Ltd.). Transducers recorded the isometric smooth muscle contractions and relaxations (BIOPAC, MP35 System Inc., COMMAT Ltd.). The tissues were suspended vertically between two platinum electrodes, and the STPT 03 Stimulator (MAY, STPT 03 Research Stimulator, COMMAT Ltd.) was used for EFS. At the start of the experiments, 2 g of pre-tension was put into the tissues and for a duration of a 1 hr tissues were washed at 15 min intervals and were let to stabilize between washings. At the beginning of the study, after the resting period, preliminary experiments were conducted and during these experiments, parameters that are suitable for EFS were determined.

***Experimental protocol***

In tissues contracted with phenylephrine (3 × 10^−5^ M), relaxation responses were taken every 2 min with a frequency of 4 Hz, an impulse train of 1 ms duration for 10 sec, and a voltage of 60 V via two parallel platinum electrodes as a stimulator. EFS-mediated relaxation responses were evaluated in the presence of atropine (10^-^^6^ M) (a nonselective muscarinic receptor antagonist) and guanethidine (10^−^^6^ M) (an adrenergic neuron blocking agent) in all groups. With atropine and guanethidine, the sympathetic and parasympathetic systems’ effects were eliminated. Guanethidine and atropine were added to the organ bath 30 min before the tissues were contracted with phenylephrine (3 × 10^−5^ M). 

***Evaluation of nicotines’ effect***

After phenylephrines’ contraction effect reached a plateau, strips were stimulated with EFS, and with the stabilization of EFS relaxation responses, 10^-4^ M of nicotine was administered to tissues then after five stimuli the EFS system was disabled (16 corpus cavernosum strips of 7 rabbits). In order to prevent any tachyphylaxis and because nicotine’s maximum effect could be seen within the first 5 stimuli, EFS was disabled after five stimuli, and tissues were washed at 15 min intervals for the duration of 1 hr. 

***Evaluation of effects of cannabinoid and vanilloid receptor antagonists on nicotine responses***

In the second part of the experimental process, strips were divided into three groups to investigate the effects of cannabinoid and vanilloid receptor antagonists on nicotine responses. AM251 (a potent CB_1_ antagonist, 10^-6^ M) (6 corpus cavernosum strips of 6 rabbits), AM630 (a selective CB_2_ inverse agonist, 10^-6^ M) (6 corpus cavernosum strips of 6 rabbits), or capsazepine (a vanilloid receptor antagonist, 3x10^-6^ M) (4 corpus cavernosum strips of 4 rabbits) were given to different tissues respectively after the resting period. Each antagonist was given only one strip of each animal. Each tissue was also incubated for 30 min with atropine (10^-6^ M) and guanethidine (10^−6^ M). After 30 min the tissues were contracted with phenylephrine (3 × 10^−5^ M) again. Then, the EFS system was enabled and with stabilization of EFS relaxation responses, nicotine administration, and five stimuli protocols were recorded again. To investigate the effect of dimethyl sulfoxide (DMSO; solvent of AM251, AM630, and capsazepine) in the experimental procedure, DMSO was given to different tissues instead of antagonists (n=2).

***Solutions and drugs used in the experiments***

The composition of the Krebs–Henseleit solution is (mM): NaCl 118; KCl 4.7; NaHCO_3_ 25; NaH_2_PO_4_**‐**2H_2_O 0.9; CaCl_2_**‐**2H_2_O 1.26; MgCl**‐**6H_2_O 0.5; and glucose monohydrate 11. Phenylephrine hydrochloride (a post-synaptic alpha-adrenergic receptor agonist), atropine sulfate (a nonselective muscarinic receptor antagonist), guanethidine monosulfate (an adrenergic neuron blocking agent), nicotine (a nonspecific nicotinic acetylcholine receptor agonist), and capsazepine (a vanilloid receptor antagonist, 3x10^-^^6^ M) were obtained from Sigma (St Louis, MO, USA) while AM251 (a potent CB_1_ antagonist) and AM630 (a selective CB_2_ inverse agonist) were purchased from Tocris (Ellisville, MO, USA). Phenylephrine hydrochloride, atropine sulfate, guanethidine monosulfate, and nicotine were dissolved in distilled water. Before use, AM251, AM630, and capsazepine were dissolved in DMSO. Stock solutions of the other drugs were prepared in distilled water. All prepared solutions were stored at −20 °C until use. The drugs were diluted with double distilled water for all processes.

***Statistical analysis***

The nicotine-induced enhancement was demonstrated as a percentage of the control responses. The maximum value of five EFS-evoked relaxation responses after nicotine treatment was used as a nicotine-induced value, while the last relaxation value before nicotine administration was used as the control value. The results were expressed as the mean ± SEM. To determine differences in the results general linear models of ANOVA were used followed by the Bonferroni *post-hoc* test. Results were considered significant when *P*<0.05. Statistical analyses were performed using the GraphPad software.

## Results

The EFS-induced relaxation responses were measured in corpus cavernosum of the rabbit. The mean magnitude of the EFS-induced relaxation responses was 961.325± 92.65 mg at a 4-Hz stimulation frequency.

***Nicotine’s effect on EFS-induced relaxation responses***

Nicotine (10^−4^ M) increased the amplitudes of EFS-induced relaxation responses (14.60% ± 2.94%; *P*=0.0002; n=16; 7 animals). 

**Figure 1:**
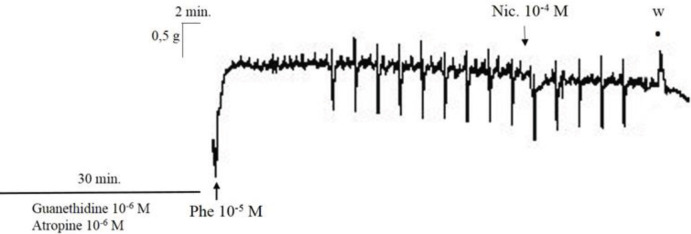
Typical recording traces showing the effect of nicotine (10-4 M)

Typical recording traces showing the effect of nicotine (10-4 M) on the EFS-induced relaxation responses in rabbit corpus cavernosum is represented in Figure 1.

These increases were reproducible and were not significantly changed during the second period of EFS after washing (n=2). 

***Cannabinoid receptor antagonist’s effect on nicotine-induced enhancement***

**Figure 2:**
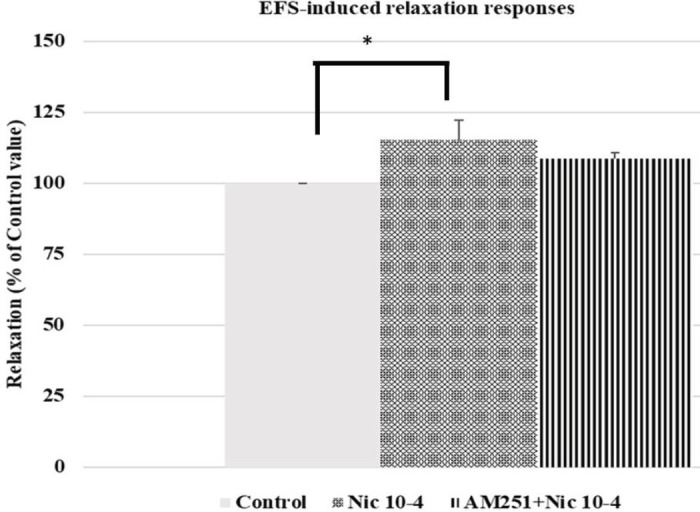
Effect of nicotine (10^-4 ^M) on EFS-induced relaxation responses (*P=0.048) and effect of AM251, a cannabinoid CB_1_ receptor antagonist (10^-6^ M), on nicotine-induced EFS relaxation response enhancement (*P*=0.611) in rabbit corpus cavernosum tissues. All points are given as the means ± SEM (n=6)

AM251 did not alter nicotine-induced EFS relaxation response enhancement significantly (nicotine 10^-4^ M enhancement: 15.20% ± 6.81%; nicotine 10^-4^ M enhancement in the presence of AM251 10^-6^ M: 8.54%±2.08%; *P*=0.611; n=6) (Figure 2).

**Figure 3:**
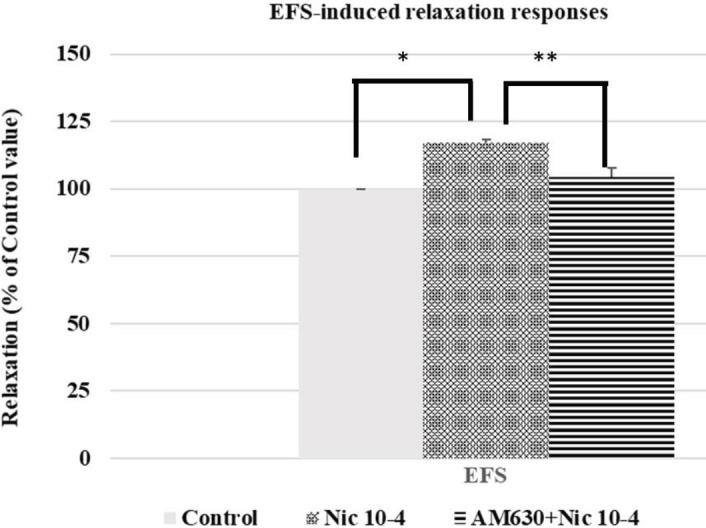
Effect of nicotine (10^-4^ M) on EFS-induced relaxation responses (*P=0.001) and effect of AM630, a cannabinoid CB_2_ receptor antagonist (10^-6^ M), on nicotine-induced EFS relaxation response enhancement (**P=0.014) in rabbit corpus cavernosum tissues. All points are given as the means ± SEM (n=6)

Nicotine-induced EFS relaxation response enhancement was inhibited by AM630 (nicotine 10^-4^ M enhancement: 17.16% ± 3.19%; nicotine 10^-4^ M enhancement in the presence of AM630 10^-6^ M: 4.44%±3.43%; *P*=0.014; n=6) (Figure 3). 

Capsazepine (a vanilloid receptor antagonist) did not alter nicotine-induced EFS relaxation response enhancement (nicotine 10^-4^ M enhancement: 13.06% ± 4.88%; nicotine 10-4 M enhancement in the presence of capsazepine 3x10-6 M: 8.99%±4.41%; *P*>0.05; n=4).

Both AM251, AM630, and capsazepine did not alter the basal tonus of the tissues and the magnitude of contraction induced by phenylephrine.

DMSO (solvent of AM251, AM630, and capsazepine) incubation did not alter nicotine-induced EFS relaxation response enhancement (n=2).

## Discussion

Nitric oxide’s role and mechanism of action on corpus cavernosum relaxation and erectile function have long been known. Although many minor factors induce relaxation responses, NO mainly mediates that response in the corpus cavernosum (1). Our previous studies demonstrated that nicotine enhanced EFS-induced nitrergic relaxation responses via nAChRs. In our previous studies, hexamethonium nearly totally inhibited nicotine-induced EFS-evoked NANC relaxation response enhancement. Those results suggested that nicotine showed those effects through nAChRs which were probably located on the nitrergic nerves of the corpus cavernosum tissue of the rabbit (3, 4). In this study at the 10^-4^ M dose, nicotine increased the amplitudes of EFS-induced relaxation responses by 14.60%. The mean magnitude of the EFS-induced relaxation responses was 961.325± 92.65 mg at the 4 Hz stimulation frequency. Nevertheless, EFS-induced relaxation responses and nicotine’s enhancement effect were higher (4). While rabbits were 3-4 months old in our previous study, rabbits were 2–3 months old in this study. Also, this study was done at the beginning of the summer in June. Both the month in which the study was conducted and the age of the animals may have influenced these results. We also showed the subunits of nAChRs that have a role in nicotine-enhanced EFS-induced relaxation responses. Subunits of alpha3–beta4, alpha4–beta2, and alpha7 of nAChRs played a role in that effect in rabbit corpus cavernosum (4). There can be a possible modulatory effect of postsynaptic released endocannabinoids on the α7 nAChRs when the relations between endocannabinoids and nicotinic receptors were considered (31). A double *in-situ* hybridization study established that α7 nAChRs and CB_1_ receptors are present simultaneously in the hippocampal interneurons (32). In rat corpus cavernosum, expression of nAChRs’ alpha7 subunit was shown. It was also demonstrated that only the alpha7 subunit is responsible for nicotine’s enhancement effect on nitrergic relaxation responses induced by EFS in that study (33). On the other hand, nicotine increases the EFS-induced responses in various tissues including the bladder. Nicotine enhanced purinergic and cholinergic transmissions through α4β2, α3β4, and α7 sub-types of nAChRs in rabbit urine bladder smooth muscle (12). 

Endocannabinoids regulate the neurotransmitter release via presynaptic cannabinoid receptors in different central and peripheral synapses (14-17). Various studies in the literature demonstrate the effects of the endocannabinoid system on the urogenital system. The functional role of cannabinoid receptors was reported in the corpus cavernosum. In that study, AM251 and AM630, but not capsazepine, inhibited EFS-induced non-nitrergic NANC relaxation responses in rabbit corpus cavernosum (5). A study reported the functional role of cannabinoid receptors in the corpus cavernosum in which AM251 and AM630, but not capsazepine, inhibited EFS-induced non-nitrergic NANC relaxation responses in rabbit corpus cavernosum (5). In a different study the presence of CB_1_ and VR_1_, but not CB_2_, receptor proteins were demonstrated in rat corpus cavernosum. The enhancement effect of anandamide on NANC mediated relaxation responses were also shown (23). The CB_1_ and CB_2_ receptors’ presence in human oocytes was also demonstrated. These receptors were detected in immature and unfertilized metaphase-II oocytes; therefore, endocannabinoids may have a role in the maturation of female gametes and fertilization (24). The correlation of anandamide with gonadotrophin and sex steroid hormones during the menstrual cycle was also demonstrated in fertile women with a normal menstrual cycle (33). Cannabinoid agonists’ possible regulatory effect on the noradrenergic responses was also shown in the rabbit vas deferens. CB_1_ receptors had a partial role in that inhibitor effect (22). 

Various studies in the literature have demonstrated the interaction between endocannabinoid and nicotinic systems, especially in the central nervous system. It was demonstrated that stress- and nicotine-related behavioral changes in mice antidepressive eﬀects of sub-chronic nicotine administration are reversed by CB_1_ and CB_2_ receptor ligands (25). The existence of CB_1_ receptors was shown in mice hypothalamus and exposure to the combination of a high-fat diet and nicotine augmented the expression of CB_1_ receptors (27). Another study showed that nAChRs might play a role in the development and phenotype of THC withdrawal. Also, nAChRs variations in different subtypes were found associated with cannabis disorder (26). Different studies demonstrated the effects of arachidonoylethanolamide (AEA) on nAChRs. It was shown that both the function of alfa4 beta nAChR in the SH-EP1 cells stably express the human α4 beta nAChR and the function of nAChRs in thalamic synaptosomes inhibited by AEA in a CB receptor-independent manner (35, 36). Another study demonstrated that AM404, an anandamide transport inhibitor, inhibited nicotine-mediated enhancement in dopaminergic transmission in the central nervous system of rats. This result may indicate that the endocannabinoid system might be a new target for the treatment of nicotine dependence (37). FAAH (Fatty acid amide hydrolase) inhibition’s enhancing effect on nicotine reward via CB_1_ receptors has been demonstrated in a different study. It has been indicated that endogenous cannabinoid tone indirectly modulates the development of nicotine addiction (38). 

In different studies, anandamide also induced relaxation responses by activating vanilloid receptors in rat corpus cavernosum and hepatic and mesenteric arteries (23, 39). Nevertheless, vanilloid receptor antagonist capsazepine did not significantly affect nicotine-induced EFS-evoked relaxation response alternation in this study. Similarly, in our previous study, capsazepine did not alter EFS-induced non-nitrergic NANC relaxation responses in rabbit corpus cavernosum (5). 

This study is the first to show the interaction between cannabinoid and nicotinic systems in the corpus cavernosum tissue. Also, while nicotine-evoked EFS relaxation response enhancement was inhibited by CB_2_ receptor antagonist AM630, CB_1_ receptor antagonist AM251, and vanilloid receptor antagonist capsazepine did not alter these responses significantly. However, in our recent study, neither cannabinoid receptor antagonists nor vanilloid receptor antagonists significantly altered the nicotine-induced EFS contraction response in rabbit bladder (unpublished data). Contrary to our results, cannabinoid inhibitors act on nAChR function in oocytes was demonstrated. In that study, anandamide, the endogenous cannabinoid, inhibited the function of alpha 7 subtypes of nAChR in Xenopus oocytes without interacting with cannabinoid receptors (29). 

In the present study, nicotine increased the amplitudes of EFS-induced relaxation responses in the corpus cavernosum, probably located on the nitrergic nerves, like in our previous studies (3, 4). In our previous studies, L-NAME nearly abolished EFS-induced relaxation of the corpus cavernosum at 4 Hz frequency (3-5) and hexamethonium subtotally inhibited nicotine-induced relaxation response enhancement (3, 4). We concluded that nicotinic acetylcholine receptors located on the nitrergic nerves of the corpus cavernosum tissue of the rabbit evoked the release of NO (3). 

EFS: Electrical field stimulation

EFS: Electrical Field Stimulation

## Conclusion

This study showed the role of cannabinoid-like endo-ligands on nicotine-induced NANC relaxation response enhancement via CB_2_ receptors but not CB_1_ and VR_1_ receptors in rabbit corpus cavernosum. The effect of endocannabinoids in NANC responses still need to be investigated in further studies. Further studies are required to identify the interaction between endocannabinoid and nicotinic systems in peripheral tissues. Finding the effects of this interaction on smooth muscle relaxation responses will be beneficial for identifying possible treatment target candidates for erectile dysfunction.

## Authors’ Contributions

IMV, GSOF Study conception and design; IMV, DSK, YO, CIA, AKK Data analyzing and draft manuscript preparation; 

IMV, GSOF, SOI Critical revision of the paper; YS Supervision of the research; IMV, GSOF, DSK, YO, CIA, AKK, SOI, YS Final approval of the version to be published (the names of all authors must be listed). 

## Conflicts of Interest

The authors declare that they have no conflicts of interest to disclose.
